# Facile Preparation and Characteristic Analysis of Sulfated Cellulose Nanofibril via the Pretreatment of Sulfamic Acid-Glycerol Based Deep Eutectic Solvents

**DOI:** 10.3390/nano11112778

**Published:** 2021-10-21

**Authors:** Weidong Li, Yu Xue, Ming He, Jiaqiang Yan, Lucian A. Lucia, Jiachuan Chen, Jinghua Yu, Guihua Yang

**Affiliations:** 1 State Key Laboratory of Biobased Material and Green Papermaking, Qilu University of Technology, Shandong Academy of Sciences, Jinan 250353, China; 17854116982@163.com (W.L.); xxyy0707@163.com (Y.X.); M17862958311@163.com (J.Y.); chenjc@qlu.edu.cn (J.C.); 2 School of Chemistry and Chemical Engineering, University of Jinan, Jinan 250022, China; chm_yujh@ujn.edu.cn; 3 Department of Forest Biomaterials, North Carolina State University, Raleigh, NC 27695, USA; 4 Department of Chemistry, North Carolina State University, Raleigh, NC 27695, USA; 5 Department of Biomedical Engineering, University of North Carolina-Chapel Hill, Chapel Hill, NC 27514, USA

**Keywords:** cellulose nanofibril (CNF), deep eutectic solvent (DES), sulfamic acid-glycerol, sulfation, characterization

## Abstract

A deep eutectic solvent (DES) composed of sulfamic acid and glycerol allowed for the sustainable preparation of cellulose nanofibrils (CNF) with simultaneous sulfation. The reaction time and the levels of sulfamic acid demonstrated that fibers could be swelled and sulfated simultaneously by a sulfamic acid-glycerol-based DES and swelling also promoted sulfation with a high degree of substitution (0.12). The DES-pretreated fibers were further nanofibrillated by a grinder producing CNF with diameters from 10 nm to 25 nm. The crystallinity ranged from 53–62%, and CNF maintained the original crystal structure. DES pretreatment facilitated cellulose nano-fibrillation and reduced the energy consumption with a maximum reduction of 35%. The films prepared from polyvinyl alcohol (PVA) and CNF showed good UV resistance ability and mechanical properties. This facile and efficient method provided a more sustainable strategy for the swelling, functionalization and nano-fibrillation of cellulose, expanding its application to UV-blocking materials and related fields.

## 1. Introduction

Cellulose nanofibrils (CNFs) are promising, high-performing materials due to their high mechanical strength and chemical versatility as well as the diversity and abundance of raw materials in which they are found [1]. CNFs have been widely used in nanocomposites, electronic and optical devices, packaging, biomedical engineering, and advanced materials [2,3,4,5,6].

CNFs are typically liberated under mechanical nano-fibrillation. Unfortunately, due to the inter- and intramolecular hydrogen bonding evident in cellulose, mechanical nano-fibrillation requires intense energy [7]. Thus, various pretreatments have been recently adopted to reduce energy consumption such as enzymes, bases, and chemical modification [8,9,10]. Such pretreatment allows for a modification of the large number of hydroxyl groups of cellulose with carboxylates (e.g., carboxymethyls) and aldehydes [11,12,13].

Sulfation is a process that introduces sulfate groups to the surface of cellulose [14]. Sulfation cellulose can be given special properties (a strong electronegativity, good solution stability, and good film formation), and is widely used in food, cosmetics, washing products, coatings, drilling and other products. The sulfation reaction in nanocellulose preparation is usually carried out to prepare cellulose nanocrystal (CNC) via sulfuric acid hydrolysis or mixed acids hydrolysis [15,16]. However, there is little information available in the literature about the sulfation of CNF. It is not suitable to modify CNF due to the high acidity of sulfuric acid, which can easily hydrolyze the amorphous region in CNF [17]. Furthermore, sulfated cellulose was reported to prepare CNF, but it was not suitable for the further mechanical treatment to prepare sulfated CNF, since the fibril structure of the prepared sulfated cellulose was destroyed or aggregated [18,19]. Post-sulfonation was also used to prepare sulfated CNF, as described by Luo et al. CNF were successfully post-sulfonated with a dimethylformamide and chlorosulfonic acid with a high surface charge while retaining a fibrous morphology [20]. However, this method was complex and the chlorosulfonic acid used was hazardous.

The deep eutectic solvent (DES) is the new generation of a green solvent composed of a hydrogen bond donor (HBD) and hydrogen bond acceptor (HBA) [21]. The melting point of the DES system is lower than that of each component [22]. It has the advantages of low cost, biodegradability, non-toxicity and renewability [23]. Recently, DES has been applied for organic synthesis, catalysis, material chemistry, and electrochemistry [24]. Lately, studies on the use of DES in cellulose functionalization (e.g., cationic functionalization, phosphine functionalization, and carbamate functionalization) and the preparation of various types of nanocellulose have emerged [25,26,27]. Sulfamic acid (amidosulfonic acid) is an inorganic solid acid formed by treating urea with a mixture of sulfur trioxide and sulfuric acid. It is a frequently used sulfation reagent with a low toxicity and low cost. Directly sulfated cellulose was successfully achieved via a sulfamic acid and urea DES to prepare nanocellulose, as described by Sirviö et al. [28]. In this study, sulfamic acid was used as the HBA, while urea was used as the HBD. Although the sulfated CNF had been prepared with a high degree of substitution, the temperature of the processed slurry was high (150 °C). Moreover, urea can engage in side reactions with cellulose, which can hinder the sulfation of cellulose [28]. In order to avoid the side reaction, it is necessary to find a reagent to replace urea in order to prepare DES with sulfamic acid and pretreat the biomass to produce sulfated CNF. However, there is little information in the literature about substituting urea in sulfamic, acid-based DES for the sulfation of cellulose. As a green and sustainable organic chemical, glycerol is a colorless, odorless, nontoxic and sweet liquid with a low price (USD 2 per kilogram) [29], which does not react with cellulose. It has been reported in the literature that glycerol is used as a hydrogen bond donor in DES to prepare CNF [30]; therefore, glycerol is a suitable alternative for urea in DES to sulfate CNF and reduce side reactions. In addition, DES provides a more sustainable strategy for the swelling because its preparation process is green, environmentally friendly, low-cost and can reduce energy consumption. Additionally, there is a lack of research focusing on reducing energy consumption in the preparation of CNF [12]. Polyvinyl alcohol (PVA) is a biodegradable polymer, which has biodegradability and a good film-forming performance [31]. In recent years, PVA has been mixed with CNF to prepare nanomembrane materials due to its good solubility and water affinity [32].

A DES synthesized by sulfamic acid (HBA) and glycerol (HBD) was used as the pretreatment medium to synthesize sulfated CNF by a supermasscolloider. In addition, the prepared CNF was used to produce polyvinyl alcohol (PVA)/CNF nanocomposite films. The sulfated fiber was analyzed through a fiber quality analysis, an elemental analysis, the degree of polymerization (DP) and Fourier transform infrared spectrometry (FTIR). The CNF was analyzed by the water retention value (WRV), atomic force microscopy (AFM), and X-ray diffraction (XRD). The mechanical energy consumption during CNF production was also analyzed. PVA/CNF nanocomposites were prepared by solvent casting, whose transparency and mechanical properties were characterized.

## 2. Materials and Methods

### 2.1. Materials

Bleached kraft poplar pulp board (with 85.05% cellulose, 14.55% hemicellulose, and 0.4% lignin) was obtained from Huatai Paper Co., Ltd. in Dongying, Shandong Province, China. It was disintegrated in water, filtered, and dried in an oven at 60 °C for 24 h. Sulfamic acid (99.5%) and glycerol (≥99%) were purchased from Shanghai Macklin Biochemical Co., Ltd. (Shanghai, China) and Sinopharm Chemical Reagent Co., Ltd. (Shanghai, China), respectively. PVA (Mw 31,000–50,000) was purchased from Shanghai Macklin Biochemical Co., Ltd. (Shanghai, China). All chemicals were of reagent grade, without any further purification. All experiments were conducted using deionized water.

### 2.2. DES Sulfated Pretreatment

The DES system (sulfamic acid with glycerol) was prepared at mass ratios of 1:3 (45.93 g and 130.69 g, respectively) [33]. The sulfamic acid and glycerol were mixed together at 90 °C with a magnetic stirrer in an oil bath until a clear liquid was formed (~two hours). The physical characteristics of DES were shown in Table 1 and were determined according to the method described by Skulcova et al. [34]. An amount of 2 g of original fibers was added to the DES (39.25 g, 58.87 g, and 78.49 g, respectively) at a constant temperature. The reaction was carried out with continuous stirring. The reaction mixture was taken out from the oil bath after a certain reaction time and cooled at room temperature for 5 min. The pretreated pulp suspensions were filtered to remove DES, physically adsorbed on the pulp surface, and washed with deionized water until the filtrate was neutral.

### 2.3. Nano-Fibrillation of Sulfated Cellulose

**Figure 1:**
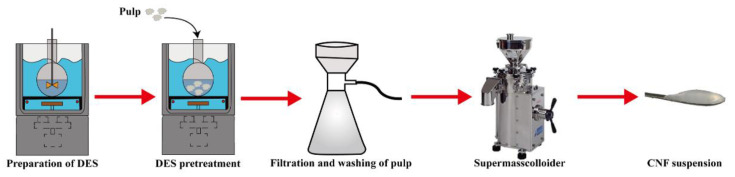
The preparation scheme of DES-untreated and DES-treated CNF.

The preparation scheme of DES-untreated and DES-treated CNF are shown in Figure 1. The DES-untreated, DES-treated pulp were disintegrated into nanofibrillation with a grinder (Supermasscolloider MKCA6-5J, Masuko Sangyo Co., Ltd., Saitama, Japan). Both DES-untreated and DES-treated pulp were diluted with deionized water to 1% consistency. The preparation process was carried out according to a method described by He et al. [7]. CNF was collected after 10 passes. The DES-untreated and DES-treated CNF samples were labeled CNF, CNF-6-1, CNF-6-1.5, CNF-9-1, CNF-9-1.5, CNF-12-1, and CNF-12-1.5, as shown in Table 2, respectively. Among them, the first digit in the code represented the sulfonic acid:cellulose mass ratio, and the second digit represented the reaction time between DES and cellulose. The energy consumption in the supermasscolloider was calculated using the amperage, flow rate, and voltage. The unit of energy consumption was kWh per kg of CNF produced on a dry basis.

### 2.4. Preparation of Neat PVA and PVA/CNF Films

PVA was dissolved in distilled water (3 wt%) and stirred for 3 h at 90 °C. After PVA solutions were cooled to room temperature, the CNF suspension was added to the PVA solutions. The dry mass of each nanocomposite was 0.6 g, the proportion of PVA was 95%, and the proportion of CNF was 5%. Finally, the dispersions were casted into polytetrafluorethylene (PTFE) Petri dishes and dried in the oven at 40 °C for 24 h. The obtained composite films were labeled PVA, PVA-CNF-6-1, PVA-CNF-6-1.5, PVA-CNF-9-1, PVA-CNF-9-1.5, PVA-CNF-12-1, and PVA-CNF-12-1.5. Among them, PVA was a film prepared from pure PVA. PVA/CNF films were composed of PVA and CNF samples (CNF-6-1, CNF-6-1.5, CNF-9-1, CNF-9-1.5, CNF-12-1, and CNF-12-1.5) which were named PVA-CNF-6-1, PVA-CNF-6-1.5, PVA-CNF-9-1, PVA-CNF-9-1.5, PVA-CNF-12-1, and PVA-CNF-12-1.5, respectively.

### 2.5. Characterizations

#### 2.5.1. Characterizations of Sulfated Pulp

Yield and fiber width were analyzed. The yield was calculated according to the absolute dry quality of pulp before and after DES treatment. The widths of original fibers and DES-treated fibers were analyzed using a fiber quality analyzer (FS5, Valmet, Espoo, Finland):

(1) $$\mathrm{yield}=\frac{m_{0}}{m}\times 100\%$$

where m_0_ is the absolute dry quality of pulp after DES treatment, and m is the absolute dry quality of pulp before DES treatment.

The average degrees of polymerization (DP) of original fibers and DES-treated pulp were obtained from the intrinsic viscosity values of the freeze-dried samples, which were dissolved in copper ethylenediamine (CED) solution according to the ISO 5351 standard. The DP was calculated from the limiting viscosity as described by Liimatainen et al. [35].

The FTIR spectra of original fibers and DES-treated pulp were performed using a FTIR instrument (ALPHA, Bruker, Karlsruhe, Germany) using oven-dried samples. The spectra were recorded in the 600–4000 cm^−1^ range, and 32 scans were taken at a resolution of 2 cm^−1^ for each sample.

The elemental analysis of DES-treated pulp before nano-fibrillation were performed with an elemental analyzer (UNICUBE, Elementar, Frankfurt, Germany). The degree of substitution (*DS*) was calculated using Equation (2) [36]:

(2) $$DS=\frac{S\times 162.15}{3206-\left(S\times 97.10\right)}$$

where *S* is the sulfur content, 162.15 mmol/g is the molecular weight of the anhydroglucose unit, and 97.10 mmol/g is the molecular weight of the ammonium sulfate group.

#### 2.5.2. Characterizations of Sulfated CNF

The water holding capacity of CNF was measured by a WRV measurement, according to the standard SCAN-C 62:00. The water holding capacity of CNF was determined using a modified WRV measurement. The *WRV* of CNF was calculated using Equation (3) [37]:

(3) $$WRV_{CNF}=\frac{WRV_{mix}-WRV_{0}\cdot 0.9}{0.1}\left(\frac{g}{g}\right)$$

where *WRV_mix_* is the *WRV* of the mixture of 90% raw pulp with 10% CNF. *WRV*_0_ is the *WRV* of the raw pulp. An average of five measurements was reported.

Atomic force microscopy (Multimode 8, Bruker, Karlsruhe, Germany) was used to scan the surface of the CNF. Before use, the aqueous suspension of 0.001% cellulose nanofibril was ultrasonically treated for 15 min. The samples were prepared by spin-coating (Easy Coater 6, Anseth Co., LTD, Beijing, China) at 3000 rpm for 30 s by depositing a drop of aqueous suspension of cellulose nanofibril (20 µL) on a freshly cleaved mica surface (TO-3P MICA, TOSAI, Tokyo, Japan) and air dried.

The zeta potential of the CNF was determined by a zeta sizer (Malvern Zetasizer Nano ZS90, Malvern Instruments Ltd., Worcestershire, UK). All samples were diluted to 0.005 wt% before analysis.

The crystallinity of the original cellulose pulp, DES-treated cellulose, and CNF were analyzed by wide-angle X-ray diffractometry (WAXD) using a Bruker D8 ADVANC X-ray diffraction (Bruker, Karlsruhe, Germany) using Cu K(alpha) radiation operated at 40 kV and 20 mA. The diffraction data were taken over a 2θ range from 10° to 45° at a scanning rate of 10°/min. The degree of crystallinity (CrI) was calculated according to Segal’s methods [38].

#### 2.5.3. Characterizations of PVA/CNF Films

Light transmittance of the neat PVA and PVA/CNF films were tested in the 200–800 nm range by using a UV-Visible spectrometer (UV-2600, Shimadzu, Kyoto, Japan).

Tensile tests were carried out by using a texture analyzer (TA.XT Express C, Stable Micro Systems, Surrey, UK). Tensile strength, Young’s modulus and elongation at break were measured. Specimens were cut into rectangles of 80 mm long and 10 mm wide. The crosshead speed used was 5 mm/min. The result was the average of five measurements.

## 3. Results and Discussion

### 3.1. Preparation of Sulfated CNF via DES

The poplar bleached pulp was pretreated by a sulfamic acid-glycerol-based DES, then the treated pulp was grinded through a supermasscollider, and sulfated CNF was finally obtained. The mass ratios between cellulose and sulfamic acid were 1:6, 1:9, and 1:12 at 100 °C; 1 or 1.5 h was selected as the pretreating time. All pretreated pulp and CNF samples were labeled, as shown in Table 2. In order to investigate the influence of different DES pretreatment conditions on the physicochemical properties of pulp fibers and final CNF products, several analyses were carried out.

### 3.2. Characteristics of Sulfated Fibers

#### 3.2.1. The Yields and Fiber Width of Sulfated Fibers

The yields of DES-treated pulp are presented in Table 3. It could be seen that reaction time and level of sulfamic acid all had obvious effects on the yields of DES-treated cellulose pulp. The yields of DES-treated cellulose pulp ranged from 80.9% to 95.2%. The yields of pulp decreased with the increase in reaction time and the level of sulfamic acid, especially pulp-12-1.5, which had the lowest yield (80.9%), likely due to the dissolution of cellulose by the formation of hydrogen bonds between DES and the hydroxyl groups of cellulose, as well as the interrupted hydrogen bonds [39].

The fiber widths of pulp fibers and DES-treated pulp are shown in Table 3. The average widths of the fibers all increased after DES pretreatment. With the same level of sulfamic acid, the average width of the fibers treated with a 1.5 h reaction time was greater than the average width treated with a 1 h reaction time. Additionally, the maximum average width was 17.4 μm (pulp-12-1.5). In the case of the mass ratio of 10:1 (sulfamic acid to cellulose), the average width of the fibers changed little with the reaction time. The increase in fiber widths was due to an increase in the swelling degree of the fibers, because DES penetrated into the cellulose fiber structure and weakened its internal hydrogen bonds [24] in accordance with Ji et al. [40].

#### 3.2.2. The Degree of Polymerization of Sulfated Fibers

The DP values of the original fibers and DES-treated pulp are presented in Table 3. The DP values of cellulose decreased with the reaction time and level of sulfamic acid. In particular, pulp-12-1.5 showed a reduction of 59% compared with the original cellulose pulp. The decrease in the DP values of cellulose was likely due to the hydrolysis of the cellulose structure induced by the sulfamic acid [41]. This was similar to the previously reported DP value of the pulp pretreated with carboxylic acid-based DES [42].

#### 3.2.3. The FTIR and Elemental Analysis Sulfated Fibers

**Figure 2:**
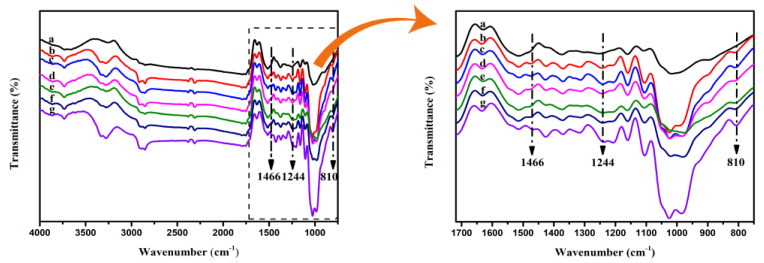
FTIR spectra of original cellulose pulp (**a**), pulp-6-1 (**b**), pulp-6-1.5 (**c**), pulp-9-1 (**d**), pulp-9-1.5 (**e**), pulp-12-1 (**f**), and pulp-12-1.5 (**g**).

The FTIR spectra of pulp fibers and DES-treated pulp are shown in Figure 2. The intensive broad band at ~3275 cm^−1^ is attributed to the OH stretching vibrations of the hydroxyl functional groups [43], whereas the peak at 2910 cm^−1^ is attributed to CH stretching. The absorption peak at 1633 cm^−1^ corresponded to the deformation vibration modes of OH from the absorbed water. The absorption peak at 1025 cm^−1^ was associated with the C-O-C pyranose ring stretching of cellulose. Two new absorption peaks at 1244 and 810 cm^−1^ could be correlated to the S=O vibration and C-O-S vibration, respectively, which indicated the formation of sulfation groups between the cellulose and sulfamic acid [44]. The absorption peak at 1466 cm^−1^ could be attributed to the deformation vibration of NH4^+^ from the ammonium salt of sulfate ester, all of which substantiated cellulose sulfation [45]. Compared with the method of Sirviö et al. [27], no side effects occurred due to the treatment in the present study.

The elemental content of sulfur is shown in Table 3. It was evident that the DES-treated pulp of the sulfur content increased which could be due to the reaction of sulfamic acid with cellulose, making sulfate groups replace the hydroxyl groups on the surface of the fibers [46]. According to Table 3, the sulfur content increased to 0.70 mmol/g with the increase in sulfamic acid and time, which might prove that a sulfation reaction between cellulose and sulfamic acid occurred. The trend of DS was consistent with the sulfur content in which the DS value of the sample pulp-12-1.5 reached a maximum of 0.12. The longer the reaction time, the better the swelling and the higher DS. This was due to the longer swelling time and the longer interactions between the cellulose fibers and DES, but caused the serious degradation of the fibers and a notable decrease in DP. The increase in DS indicated that the reaction was more intense, and it would be accompanied by a decrease in DP.

### 3.3. Characterization of Cellulose Nanofibril

#### 3.3.1. Water Retention Value and Energy Consumption of Cellulose Nanofibril

**Figure 3:**
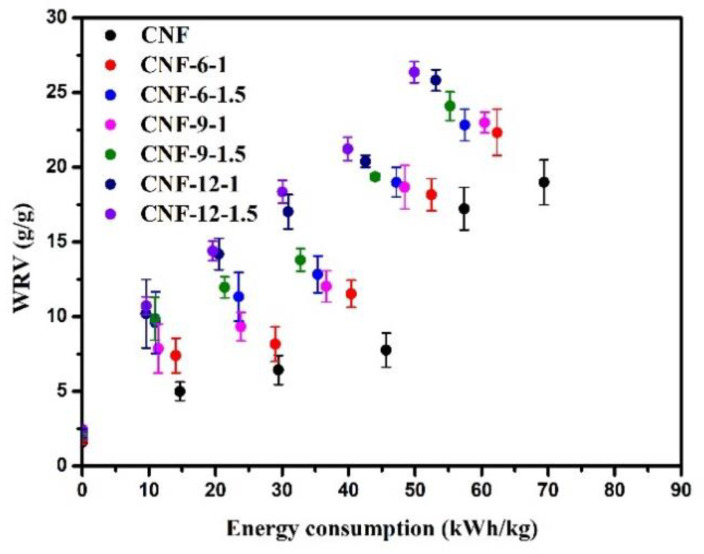
Water retention value (WRV, g/g) of CNF samples as a function of energy consumption (kWh/kg) in the supermasscolloider.

The water retention value (WRV) of the CNF samples as a function of energy consumption in the supermasscolloider is shown in Figure 3. The curves were fitted and the R^2^ were in the range of 0.97–0.99. The WRV increased with fibrillation, consistent with previous studies [47]. It was reported that the external cell wall, the primary (P), and the first secondary (S1) layers of the cellulose fibers were gradually stripped from the fiber surface, and the thick secondary cell wall was exposed by the refining cellulose fibers with a disk refiner [48]. Similar to refining, the fracture and dissociation of the fibers happened under the mechanical grinding by the supermasscolloider, which resulted in the improvement of the inter-fiber bonding and water retention capacity [49]. The high water retention value indicated that the hydrophilicity of the fibers was high, and that the fibers could swell and became easier to fibrillate under the action of mechanical shear force. At the same WRV, the energy consumption of CNF from DES-treated cellulose pulp was lower than that of the CNF prepared from the original cellulose pulp, which indicated that the pretreatment of sulfamic acid and glycerol DES could reduce energy consumption, and cellulose was more easily fibrillated. This was explained by DES penetrating into the cellulose fiber structure, which weakened the connection between the fibers, promoting fiber fracture and unraveling. Compared with the energy consumption of CNF prepared from raw materials, the energy consumption of CNF pretreated with DES had a maximum reduction of 35% (CNF-12-1.5). The DES treatment could reduce energy consumption, especially with the extension of the reaction time and the increase in sulfamic acid.

#### 3.3.2. The Preparation Mechanism of Sulfated Cellulose Nanofibril

**Figure 4:**
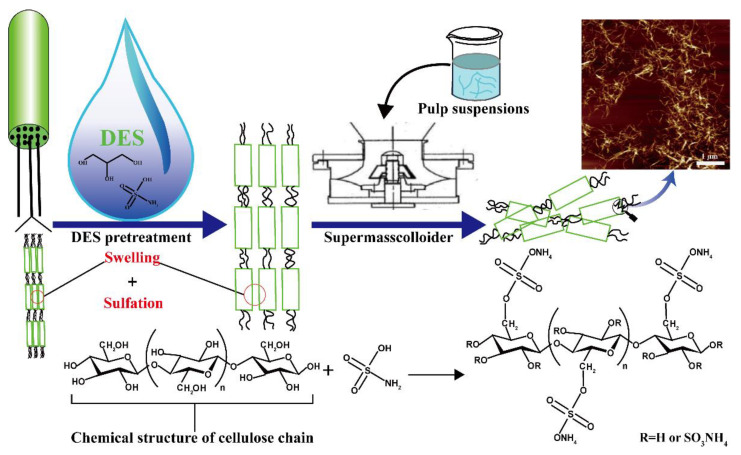
Schematic diagram for preparation of CNF through DES pretreatment.

A representation of CNF preparation with sulfamic acid and glycerol DES pretreatment is shown in Figure 4. Sulfamic acid was used as a hydrogen bond donor and glycerol as a hydrogen bond acceptor. The original fibers were treated with DES for 1 or 1.5 h to make the fiber swell and sulfate the hydroxyl of the cellulose. The glycerol entered the cellulose fiber interior to disrupt the inter- and intra-molecular hydrogen bonds between the fibers, resulting in swelling [50]. The swelling caused a greater surface area, revealing more hydroxyl groups [51]. Sulfamic acid was a zwitterionic tautomer, expressed as H_2_NSO_2_(OH) in the form of H_3_NSO_3_ [52]. The sulfation reaction led to the replacement of the hydroxyls by the sulfate group. The swelling of glycerol facilitated the penetration of sulfamic acid and the reaction with the hydroxyl groups and promoted nano-fibrillation. Utilizing the deep eutectic solvent system allowed for the simultaneous swelling and ensuing sulfation. Moreover, glycerol did not engage in side reactions, as was the case with urea. After DES pretreatment, the cellulose fiber was further fibrillated by the supermasscolloider. Friction and tearing occurred under the extrusion pressure of DES-prepared cellulose fiber bundles resulting in a reduction in the radial size of the expanded cellulose fiber bundles [53]. Finally, the cellulose fibers were separated by the interaction of fiber and mechanical grinding to obtain sulfated CNF.

#### 3.3.3. The Morphology and Size Dimensions of Cellulose Nanofibril

**Figure 5:**
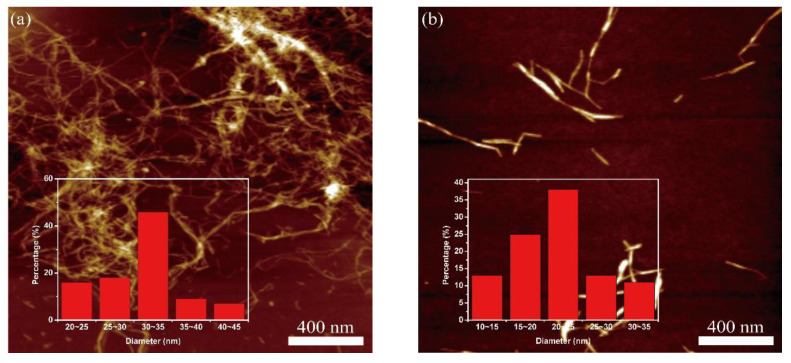

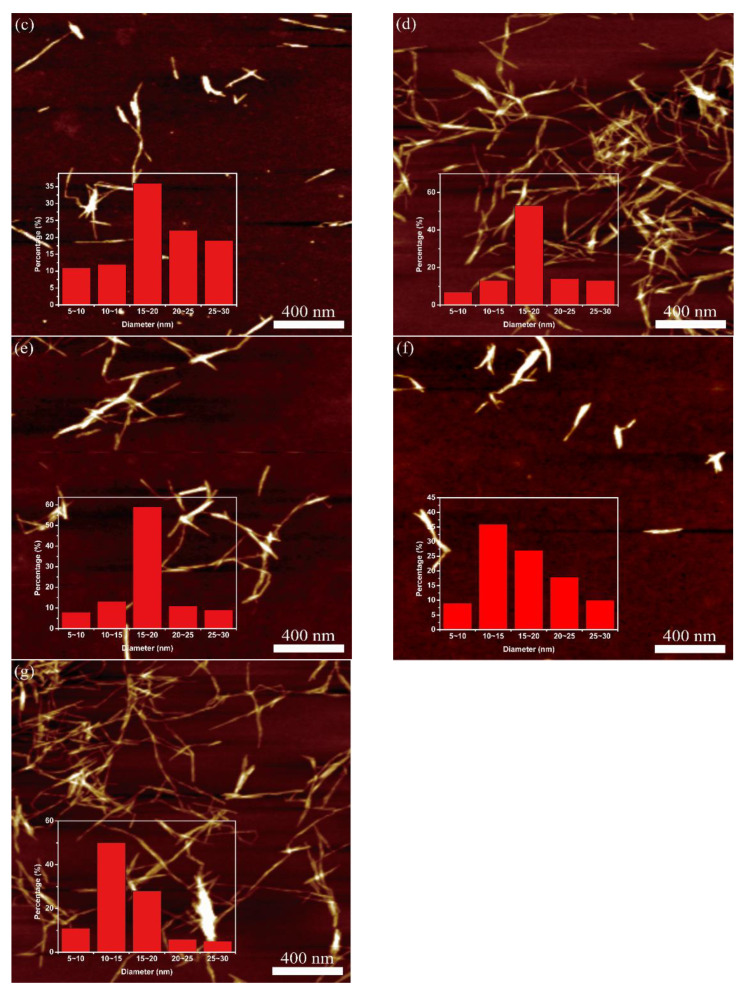
AFM topography images, and diameter distribution histograms of (**a**) CNF, (**b**) CNF-6-1, (**c**) CNF-6-1.5, (**d**) CNF-9-1, (**e**) CNF-9-1.5, (**f**) CNF-12-1, and (**g**) CNF-12-1.5.

The morphology and dimensions of the nanofibril samples were characterized by AFM. Figure 5a is the AFM topograghy image and diameter distribution histogram of CNF from the original cellulose pulp; typical CNF is visible with interwound networks and a diameter of CNF in the range of 30–35 nm, attributed to the mechanical shearing force of the supermasscolloider that promoted the fracture of the cellulose chain and nano-fibrillation. Figure 5b–g illustrate the AFM topograghy image and diameter distribution histogram of CNF-6-1, CNF-6-1.5, CNF-9-1, CNF-9-1.5, CNF-12-1 and CNF-12-1.5, respectively. The shorter fibrils compared with CNF from the original cellulose pulp were visible, with diameters in the range of 20–25 nm, 15–20 nm, 15–20 nm, 15–20 nm, 10–15 nm, and 10–15 nm, respectively. This result was related to the interaction between sulfamic acid and the hydroxyl groups to graft the sulfate group onto the surface of cellulose, thereby weakening the hydrogen bond of the fiber. Meanwhile, the effect of mechanical shear force cut the fibers swollen in the DES pretreatment process into shorter CNFs, which could be used for applications in composites requiring robust mechanical properties.

#### 3.3.4. The Zeta Potential and Crystallinity of Cellulose Nanofibril

The zeta potential value of CNF suspension is shown in Table 3. Zeta potential could be used to indicate the degree of electrostatic repulsion between similar charged particles in the dispersion [54]. The zeta potential value of CNF suspension had a potential range between −18.3 mV and −35.2 mV, which was in compliance with the values measured in recent studies [55,56]. Compared with the CNF obtained from the original fibers, the zeta potential value of CNF obtained from DES-pretreatment increased. The maximum increase in CNF-12-1.5 was 92% compared with the original CNF because sulfamic acid introduced sulfate groups to the surface of the fibers with negative charges and increased the anion zeta potential. Generally, the zeta potential values between −30 mV and −40 mV showed a moderate stability [57]. Principally, the high value of zeta potential indicated a high degree dispersion of CNF. The negative zeta potential caused by sulfation led to electrostatic repulsive forces and well-dispersed CNF suspensions.

**Figure 6:**
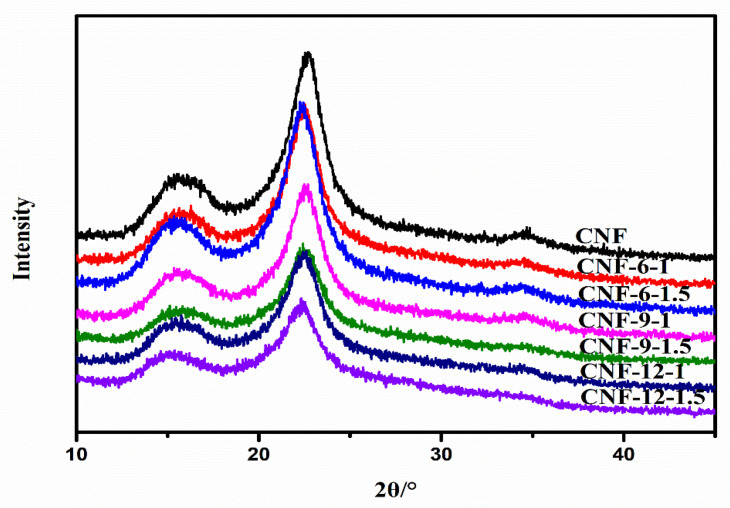
X-ray diffraction curves of original fibers and DES-treated pulp after nano-fibrillation.

Each sample in Figure 6 exhibited three typical diffraction peaks at 2θ = 16.3°, 22.6°, and 34.8°, characteristic of cellulose I allomorphism, attributed to the 110, 200, and 004 crystalline planes, respectively [58]. This result revealed the DES modification in which the consequent mechanical nano-fibrillation did not change the crystal structure. The CrI values were 64.0%, 61.7%, 60.2%, 57.6%, 54.5%, 54.8%, and 53.3%, corresponding to CNF, CNF-6-1, CNF-6-1.5, CNF-9-1, CNF-9-1.5, CNF-12-1, and CNF-12-1.5, respectively, as summarized in Table 3. The CrI values of CNF prepared from DES-treated pulp all decreased compared with the CNF prepared from the original fibers, this might imply that the sulfate groups on the sulfated CNF were detected as amorphous regions [59]. In addition to the influence of sulfate groups, the mechanical grinding force disrupted the hydrogen bonds between the crystalline and amorphous regions and ultimately led to a decrease in CrI.

### 3.4. Characterization of Neat PVA and PVA/CNF Films

**Figure 7:**
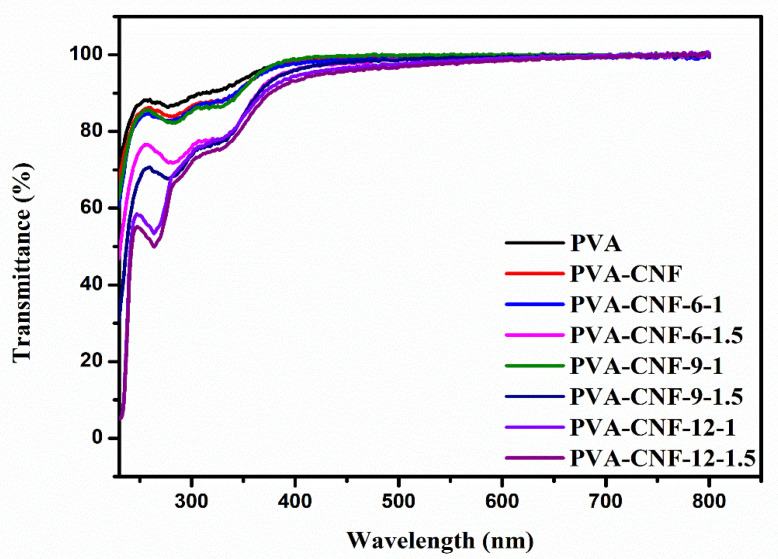
UV-Visible transmittance of neat PVA and PVA/CNF films.

As shown in Figure 7, in the UV spectrum and the transmittance of PVA/CNF films decreased compared to the neat PVA film. For example, at 277 nm, the UV light transmittance of the neat PVA film was 86%, but the UV light transmittance of the samples PVA-CNF, PVA-CNF-6-1, PVA-CNF-6-1.5, PVA-CNF-9-1, PVA-CNF-9-1.5, PVA-CNF-12-1 and PVA-CNF-12-1.5 were 84%, 82%, 72%, 81%, 68%, 64% and 61%, respectively. This indicated that the UV resistance ability of the PVA/CNF films improved, consistent with Niu et al. [60]. The addition of sulfated CNF improved the UV resistance of the film more than the addition of unfunctionalized CNF in the PVA matrix. The UV resistance ability of the sample PVA-CNF-12-1.5 was the most effective. In the visible light region (400–800 nm), the addition of CNF had a notable effect on the total transmittance. The transparency of the film could be assessed by UV-Visible spectrometry. The transmittance of all samples was almost same at the wavelength of 700 nm, which indicated that the addition of CNF had little effect on the transparency of the visible light of PVA.

**Figure 8:**
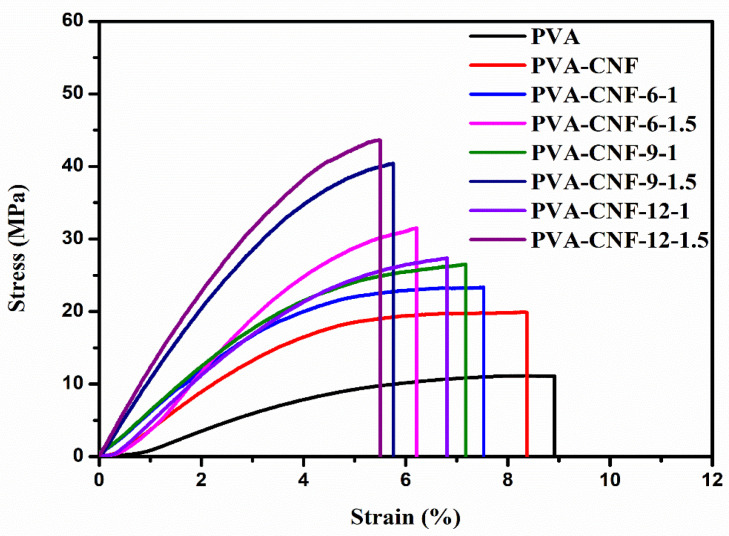
Stress–strain curves of neat PVA and PVA/CNF films.

The stress–strain curves and tensile properties from the tensile measurements of PVA/CNF films are shown in Figure 8 and Table 4. The addition of CNF improved the tensile strength and the elastic modulus was compared to the neat PVA film, especially in PVA-CNF-12-1.5 where the maximum tensile strength and Young’s Modulus increased to 44 ± 3 MPa and 1629 ± 128 MPa, respectively. Because of the high aspect ratio and high elastic modulus of cellulose, the Young’s modulus and tensile strength of PVA/CNF composite films improved. However, the elongation at the break decreased after adding CNF that was ascribed to the intramolecular and intermolecular hydrogen bonds formed between PVA and CNF [61]. The improvement effect of CNF with DES treatment was more effective than without DES treatment. The amount of sulfamic acid used in the pretreatment of CNF influenced the mechanical properties of the composite films. At the same reaction time, Young’s modulus and the tensile strength increased with the increase in the content of sulfamic acid, which could be due to that the introduction of sulfate groups on the CNF surface, and this caused less aggregation in the PVA matrix under the action of electrostatic repulsion, which enhanced the contact between the CNF surface and the PVA matrix and made it easier to form hydrogen bonds between the two [62].

## 4. Conclusions

Sulfamic acid-glycerol DES was successfully applied to prepare sulfated CNF combined with mechanical disintegration. The DES pretreatment was carried out with a sulfamic acid:glycerol mass ratio of 1:3 under 100 °C for 1 or 1.5 h. The results showed that the fiber width of the pulp fibers increased by 16–30% after DES pretreatment, which indicated that the fibers were swollen. Meanwhile, sulfation was corroborated according to FTIR spectra and elemental analysis, in which the maximum DS of the sulfated cellulose was 0.12. The sulfamic acid-glycerol DES may swell and sulfate fibers in one-step, in which swelling also promoted sulfation. After further nano-fibrillation, the sulfated CNF was obtained in the diameter range 10–25 nm. Moreover, DES pretreatment facilitated cellulose nano-fibrillation and reduced energy consumption, with a maximum reduction of 35%. The obtained CNF maintained the original crystal structure, but the crystallinity was reduced to 53% due to the introduction of the sulfate groups. In addition, the prepared PVA/CNF composite film showed excellent UV resistance ability and tensile strength.

## Figures and Tables

**Table 1:** Physical characteristics of sulfamic acid and glycerol DES at 25 ± 2 °C.

pH	Viscosity (mPa·s)	Melting Point (°C)
2.36 ± 0.03	1389 ± 2	−63.6 ± 0.1

**Table 2:** Pretreatment conditions of cellulose in sulfamic acid and glycerol DES.

Sample	Sample	Sulfamic Acid:Cellulose Mass Ratio	Time (h)
Pulp-6-1	CNF-6-1	1:6	1
Pulp-6-1.5	CNF-6-1.5	1:6	1.5
Pulp-9-1	CNF-9-1	1:9	1
Pulp-9-1.5	CNF-9-1.5	1:9	1.5
Pulp-12-1	CNF-12-1	1:12	1
Pulp-12-1.5	CNF-12-1.5	1:12	1.5

**Table 3:** Characterization of pulp fibers and CNFs.

Sample	Yield (%)	Width (µm)	DP	S (mmol/g)	^1^ DS	Sample	ZP (mV)	CrI (%)
Original cellulose pulp	-	13.4	1012	0.00	0.00	CNF	−18.3	64.0
Pulp-6-1	95.2	15.5	638	0.18	0.01	CNF-6-1	−29.4	61.7
Pulp-6-1.5	86.5	15.6	517	0.50	0.09	CNF-6-1.5	−30.2	60.2
Pulp-9-1	94.9	15.8	517	0.20	0.03	CNF-9-1	−31.0	57.6
Pulp-9-1.5	84.5	17.3	460	0.53	0.09	CNF-9-1.5	−31.8	54.5
Pulp-12-1	92.2	16.9	513	0.28	0.05	CNF-12-1	−31.3	54.8
Pulp-12-1.5	80.9	17.4	412	0.70	0.12	CNF-12-1.5	−35.2	53.3

^1^ Calculated from the elemental content of sulfur.

**Table 4:** Mechanical properties of neat PVA and PVA/CNF films.

Sample	Young’s Modulus (MPa)	Maximum Tensile Strength (MPa)	Elongation at Break (%)
PVA	221 ± 74	11 ± 3	8.9 ± 0.4
PVA-CNF	444 ± 68	19 ± 2	8.4 ± 0.2
PVA-CNF-6-1	569 ± 59	23 ± 2	7.5 ± 0.2
PVA-CNF-6-1.5	602 ± 81	31 ± 4	6.2 ± 0.1
PVA-CNF-9-1	576 ± 93	26 ± 3	7.2 ± 0.3
PVA-CNF-9-1.5	1493 ± 101	40 ± 5	5.8 ± 0.1
PVA-CNF-12-1	584 ± 76	27 ± 7	6.8 ± 0.8
PVA-CNF-12-1.5	1529 ± 128	44 ± 3	5.5 ± 0.4

## Data Availability

The data presented in this study are available on request from the corresponding author.
